# Metal Ions Released from Fixed Orthodontic Appliance Affect Hair Mineral Content

**DOI:** 10.1007/s12011-014-0152-z

**Published:** 2014-10-19

**Authors:** Marcin Mikulewicz, Paulina Wołowiec, Bartłomiej Loster, Katarzyna Chojnacka

**Affiliations:** 1 Department of Dentofacial Orthopaedics and Orthodontics, Division of Facial Abnormalities, Medical University of Wrocław, Krakowska 26, Wrocław, 50-425 Poland; 2 Institute of Inorganic Technology and Mineral Fertilizers, Wrocław University of Technology, Wrocław, Poland; 3 Department of Orthodontics, Medical College, Jagiellonian University, Wrocław, Poland

**Keywords:** Chromium, Fixed orthodontic appliance, Hair mineral analysis, Kinetics, Nickel

## Abstract

The objective was to evaluate metal ion accumulation in hair of patients undergoing orthodontic treatment with fixed appliances in time. The patients (*N* = 47) participated in a questionnaire survey. Hair sampling was performed at the beginning and in the 4th, 8th, and 12th month of the treatment. The content of metals (Cr, Ni, Fe) in hair was analyzed by ICP–OES equipped with USN nebulizer. The peak release of Cr and Fe occurred after 4 months of the treatment, and the peak release of Ni gradually increased throughout the whole year of the therapy. During 1 year treatment, an average accumulation of metals in hair tissue was 7.42 ± 14.19 μg of Ni, 8.94 ± 13.1 μg of Cr, and 131 ± 279 μg of Fe. The mean content of Cr was higher than the 90th percentile value for this element. The upper limit of literature reference ranges for Cr, Ni, and Fe in hair was not exceeded. The value of exposure (kinetics and dose) of orthodontic patients to metal ions released from orthodontic appliances can be assessed by hair mineral analysis. The content of Cr was statistically significantly higher during the treatment than before the beginning of therapy.

## Introduction

An increasing demand for biocompatibility testing of medical devices has led to the application of different evaluation methods. In contemporary dentistry, applied materials are being introduced to the market and frequently one material promptly replaces another, which entails questions about long-term biocompatibility [1]. Of invasive and non-invasive accessible biomarkers of exposure, the latter seem to be the most practical. Hair mineral analysis as a biomarker has been used for several years [2]. The collected material is digested and after solubilization undergoes multielemental analysis by various spectroscopic techniques (e.g., ICP–OES (inductively coupled plasma–optical emission spectroscopy), ICP–MS (inductively coupled plasma–mass spectroscopy), AAS (atomic absorption spectroscopy)). The sensitivity of the instruments enables determination of elements even at trace levels [3].

Usually, orthodontic treatment with fixed appliances lasts for approximately 2–3 years. Most of the patients are youngsters or adolescents. Some parts of a removable appliance and all parts of a fixed appliance are manufactured from alloys which contain, inter alia, nickel and chromium [4]. Those metals are well known as sensitizing and mutagenic agents [5, 6]. This shows that it is important to assess biocompatibility of orthodontic appliances.

In the evaluation of biocompatibility of materials used in orthodontics, chemical techniques are indispensable. In order to assess the real exposure of patients to the chemical elements released from metal alloys, tests on patients undergoing orthodontic therapy are essential. This enables monitoring side effects of orthodontic treatment, in particular the exposure to the products of corrosion of stainless steel. In the current literature, the studies on the release of metal ions concern in vitro [7] and in vivo: saliva [8, 9], blood [10], epithelium cells [11, 12], urine [13], and hair [14] were reported. The content of the elements in various matrices reflects different windows of detection: for urine and saliva (36–72 h, the measure of acute exposure), which is similar as for blood [2]. Since patients experience pain as blood and epithelium cells are being sampled, it is difficult to meet the monitoring study requirements [15]. Element levels in hair reflect a long-term (chronic) exposure, and the quantity of excreted elements by this route can be considered as an indirect measure of exposure [16]. In a long-term exposure (which is the case of orthodontic treatment), the question how to select the best biomarker for the specific clinical situation arises. Because of the wide window of detection, the concept of using hair as a non-invasive matrix to investigate the release of metal ions from orthodontic appliance seems to be reasonable [2].

The objective of the present work was to evaluate metal ions released to human hair in patients undergoing orthodontic treatment with fixed appliances in time.

## Materials and Methods

Research was performed in accordance with the principles laid down in the Helsinki Declaration. The study was carried out with the approval of the Ethical Committee of Wroclaw Medical University (KB-400/2010). The material (hair) was collected from a group of 47 patients (volunteers) (31 females, 16 males; average age was 17.2 ± 6.6 years; the minimum age was 11 and the maximum was 31), treated at the Orthodontic Department of the Faculty of Medicine, the Jagiellonian University in Cracow, Poland. The patients signed the agreement to participate in the study. The inclusion criteria for the study were lack of previous orthodontic treatment and generally good health. Patients were treated with the use of a full (upper and lower arch) orthodontic fixed appliance: brackets (Victory series; SS, 3M Unitek, Monrovia, Calif, USA), bands (SS, 3M Unitek, Monrovia, Calif, USA), and wires (NiTi, SS, 3M Unitek, Monrovia, Calif, USA). The sampling strategy was as follows: before the treatment (as a control) and on 4th, 8th, and 12th month of the treatment (average sampling interval 4.14 ± 1.87 months). The patients were instructed on how to collect the samples (3–4 cm of the hair strand measured from the scalp was sampled, shortly after the hair had been washed). Each patient received a sample container and a shampoo (baby shampoo, containing no functional components). It was explained to the patients that the meaningful and interpretable result of the hair mineral analysis could only be obtained when the specified procedures for washing hair were complied with.

### Questionnaire Survey

Before the treatment, the patient and the orthodontist were asked to fill out the electronic questionnaire: the patient about his lifestyle and possible sources of exposure, the orthodontist about the types of used appliances. The questionnaire included questions on lifestyle habits: general information (age, sex), hair (type, color), medical history, supplementation (vitamin–mineral preparations), dietary habits (fruits, vegetables, meat, fish, mushrooms), environmental exposure (place of living), and others (smoking, jewelry). This enabled pointing out the sources of exposure to toxic metals and identifying the sources of exposure stemming from the patient’s diet.

The material was sent (the hair samples were coded) to a specialized laboratory where hair samples underwent purification from organic components and multielemental analyses by ICP–OES.

### Analytical Methods

The samples (0.5 g) were decomposed with concentrated nitric acid—69 % (*m*/*m*) (5 mL), spectrally pure (Suprapur, Merck, Darmstadt, Germany), in Teflon bombs in the Milestone Start D microwave oven (Sorisole, Italy). Such parameters of decomposition were selected as to achieve the complete mineralization of the samples. After mineralization, the samples were diluted with double demineralized water (Millipore Simplicity, Molsheim, France) to 50 g. Next, multielemental analyses by ICP–OES Varian-Vista MPX (Australia) with ultrasonic nebulizer CETAC U5000AT+ were performed. The analyses were carried out in the quality management system (PN-EN ISO/IEC 17025:2005; accreditation number AB 696 PL PCA). The analyses were repeated three times. The quality control of the analytical process was achieved by the application of Certified Reference Material—Human Hair NCS ZC81002 (China National Analysis Centre). All the hair samples were sent to the laboratory in one batch and analyzed together under a single calibration. Single element laboratory standards from Ultra Scientific were used for determination of Cr, Fe, and Ni. The calibration curve included the following concentrations: 0.01, 0.025, 0.05, 0.1, 0.5, 1, and 5 mg/L. The laboratory used quality control procedures according to the implemented Quality Assurance System.

### Estimated Hair Mass Growth Rate

An adult has approximately 100,000 hairs on the scalp [17], and the average growth rate is 1 cm/month [2]. It was calculated that 1 cm of single hair of a Caucasian individual has a mass of 0.0518 mg (200 hairs with the length of 10 cm were weighed). Estimated hair mass growth rate was 5.18 g/month, as shown by the following equation:

$$ {r}_{\mathrm{hair}}\left(\frac{g}{\mathrm{month}}\right)={r}_{\mathrm{hair}}\left(\frac{\mathrm{cm}}{\mathrm{month}}\right)\times \mathrm{hair}\ \mathrm{count}\left(100,000\right)\times {m}_{\left(1\ \mathrm{cm}\ \mathrm{hair}\right)}\left(0.0518\ \mathrm{mg}\right) $$

where

*r*_hair_: Rate of hair growth
hair count: The average number of hair on human head
*m*_(1 cm hair)_: The average mass of a single hair strand, 1-cm length

Hair mass growth rate was calculated to determine the mass of nickel/chromium/iron accumulated in hair tissue during 1 month. The rate of hair growth (kg/month) was multiplied per the content of element (mg/kg), yielding the rate of accumulation (mg/month).

### Statistical Methods

The results were elaborated statistically in *Statistica* ver. 10.0. Descriptive statistics (means, standard deviations, and medians) were reported. Normality of distribution of the results was investigated by the Shapiro–Wilk test. Statistical tests were performed by the analysis of the variance with repeated measurements. The ANOVA analysis was performed to determine the expected equivalent boundary, with the statistical significance set at *p* ≤ 0.05 and significance being *p* ≤ 0.1. Then, a post hoc analysis was undertaken with the Tukey test for the groups of different samplings as the effect. The non-parametric Mann–Whitney *U* test was used to investigate the effect of the questionnaire survey on the level of Cr and Ni in hair before the beginning of treatment. The statistically significant parameters were considered in a multiple regression analysis. Statistical significance was set at *p* ≤ 0.1.

## Results

The power test analysis was performed by univariate analysis of variance. The purpose of this analysis was to compare the mean value of four independent groups of results. Thus, the hypothesis of their equality was tested and confirmed. Univariate analysis of variance was performed at the level of type I error (*α*) equal to 0.05. It was assumed that root-mean-square standardized effect (RMSSE) in the population was 0.29008. For the sample size used (*N* = 47) in the experiment, at the level of significance set at 0.5 %, the power of the test was 82.70 %. The test was considered strong.

**Fig. 1:**
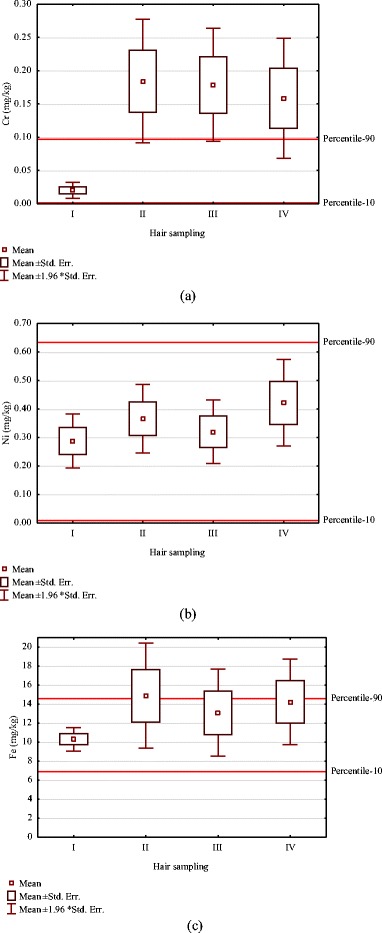
The content of **a** Cr, **b** Ni, and **c** Fe in hair of patients in time during 1 year of orthodontic treatment

The sample loss during the experiment was 17 % (8 patients). The content of Ni and Cr substantially increased during the treatment (Table 1), in particular during the first months after the insertion of orthodontic appliance. The differences were statistically significant for Cr. For Ni and Fe, they were not statistically significant (Fig. 1). The ranges between 10th and 90th percentile for the studied group, before the beginning of the orthodontic treatment, were evaluated (Table 2) and marked on Fig. 1. Table 1 shows that there were no statistically significant differences for Ni, although the content of this element increased over 46 % in the final sampling.

**Table 1:** Contents of elements in hair samples, mg/kg

Metal	Hair I			Hair II			Hair III			Hair IV		
	*N* = 47			*N* = 47			*N* = 45			*N* = 39		
	Mean	SD	Median	Mean	SD	Median	Mean	SD	Median	Mean	SD	Median
Cr	0.0201^a,b,A^	0.0430	0.00100	0.184^a^	0.326	0.0218	0.179^A^	0.291	0.0284	0.158^b^	0.287	0.00717
Ni	0.288	0.334	0.198	0.366	0.418	0.217	0.320	0.381	0.206	0.422	0.483	0.345
Fe	10.3	4.3	9.301	14.9	19.3	12.0	13.1	15.7	10.4	14.2	14.3	10.3

Hair I—before the treatment, Hair II—4th month of the treatment, Hair III—8th month, Hair IV—12th month

Analysis of variance with repeated measurements, Tukey test: ^A^(*p* ≤ 0.05), ^a,b^(*p* ≤ 0.1)

**Table 2:** Contents of chromium and nickel in hair collected before treatment, mg/kg

	Mean	SD	Median	10–90 Percentile	*p*
Cr					
Total, *N* = 47	0.0201	0.0430	0.00100	0.00100–0.09719	–
Females, *N* = 31	0.0212	0.0422	0.00100	0.00100–0.09719	0.907
Males, *N* = 16	0.0178	0.0465	0.00100	0.00100–0.02910	
<18 years, *N* = 30	0.0210	0.0436	0.00100	0.00100–0.11439	0.842
>18 years, *N* = 17	0.0187	0.0433	0.00100	0.00100–0.04946	
Ni					
Total, *N* = 47	0.275	0.325	0.197	0.00843–0.57395	–
Females, *N* = 31	0.300	0.373	0.197	0.00945–0.57395	0.954
Males, *N* = 16	0.220	0.166	0.205	0.00500–0.41510	
<18 years, *N* = 30	0.312	0.330	0.230	0.0423–0.6038	0.994
>18 years, *N* = 17	0.211	0.316	0.144	0.00616–0.41510	
Fe					
Total, *N* = 47	13.2	14.6	10.4	6.31–18.4	–
Females, *N* = 31	9.90	3.01	9.34	6.88–13.0	–
Males, *N* = 16	11.6	6.28	9.14	7.13–20.8	
<18 years, *N* = 30	10.9	5.19	9.19	6.81–20.1	–
>18 years, *N* = 17	9.74	2.11	9.34	7.13–12.8	

Mann–Whitney *U* test was used

On the basis of probability *p*, it was evaluated whether the distribution of the variable (Cr, Fe, and Ni content) can be considered normal. In our case, *p* = 0.0000 was less than the significance level *α* = 0.05; therefore, the hypothesis of the normal distribution of results was rejected. The data were log transformed, which also yielded a not normal distribution. Consequently, a non-parametric test was used to identify the significance of the results. Table 2 shows median values. Mean values and standard deviations were given for information. For Cr, the median was set at the level of 0.00100 mg/kg because before the orthodontic treatment in 33 out of 47 samples, the content in the analyzed sample was below the lower limit of detection (LLD; Table 2). After the treatment, the content of Cr substantially increased.

The statistical significance of differences was found for Cr between the samplings. After 4 months of the treatment, the content of Cr increased over nine times and then gradually decreased. The explanation could be the formation of the oxide layer which protects from further release. The decrease in the level of Cr in hair might result from previous detoxication. The differences were statistically significant. The release of Ni to human hair tissue was not as strong: a 27 % increase after 4 months, 46 % after 1 year. Fe is the main component of stainless steel; however, it is not of toxicological concern. The increase in Fe level was by 45 % during 4 months, with the decrease over time being down to 38 % after 12 months.

**Fig. 2:**
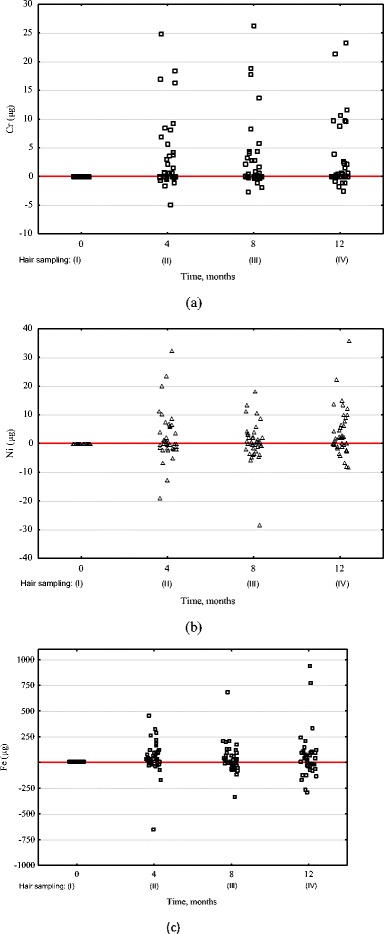
Accumulation of **a** Cr, **b** Ni, and **c** Fe after 1 year of orthodontic treatment in hair tissue of patients in time (as related to sampling I of individual patients)

**Fig. 3:**
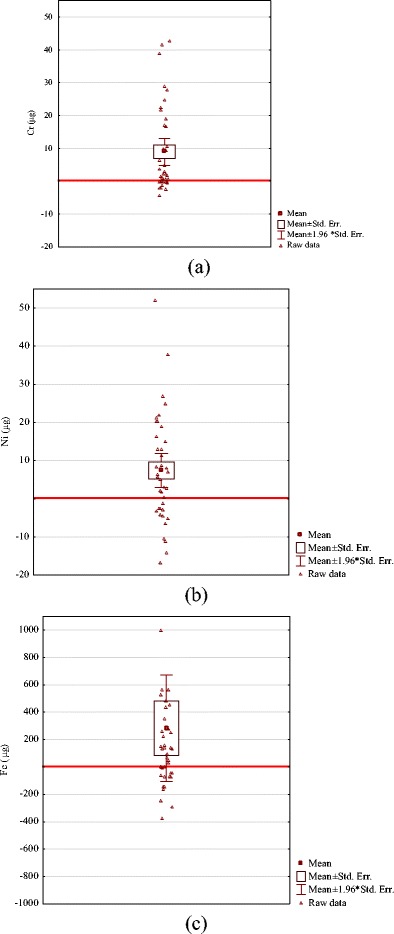
Accumulation of **a** Cr, **b** Ni, and **c** Fe in hair tissue after 12 months of orthodontic treatment

The mass of released Cr, Ni, and Fe ions that was transferred to hair tissue was calculated on the basis of empirically determined hair mass growth rate (5.18 g/month). This enabled us to determine the mass of Cr, Ni, and Fe excreted to hair of an individual patient, as compared to the mass at the starting point of the therapy within sampling intervals (Fig. 2), as well as to the accumulation after 1 year of orthodontic treatment in hair (Table 3, Fig. 3).

**Table 3:** Released mass of chromium, nickel, and iron after 1 year of the treatment, μg/year (*N* = 39)

	Mean	SD	Min	Max
Cr	8.94	13.1	−4.44	42.7
Ni	7.42	14.19	−16.7	51.9
Fe	131	279	−371	999

The accumulation after 1 year of orthodontic treatment (*m*_12 months_) was described with the following equation:

$$ {m}_{12\ \mathrm{months}}=\frac{\left({C}_{\mathrm{I}\mathrm{I}}-{C}_{\mathrm{I}}\right)\cdot {r}_{\mathrm{hair}}\left(\frac{\mathrm{g}}{\mathrm{month}}\right)}{\mathrm{time}\left(4\ \mathrm{months}\right)}+\frac{\left({C}_{\mathrm{I}\mathrm{I}\mathrm{I}}-{C}_{\mathrm{I}\mathrm{I}}\right)\cdot {r}_{\mathrm{hair}}\left(\frac{\mathrm{g}}{\mathrm{month}}\right)}{\mathrm{time}\left(4\ \mathrm{months}\right)}+\frac{\left({C}_{\mathrm{I}\mathrm{V}}-{C}_{\mathrm{I}\mathrm{I}\mathrm{I}}\right)\cdot {r}_{\mathrm{hair}}\left(\frac{\mathrm{g}}{\mathrm{month}}\right)}{\mathrm{time}\left(4\ \mathrm{months}\right)}\left(\frac{\mathrm{mg}}{12\ \mathrm{months}}\right) $$

where

*m*_12 months_ is the accumulation of metal ions after 1 year of orthodontic treatment.

This mass is referring to the sum of all measurements in 4-month intervals, as referred to the previous. Baseline plotted at *m*_Cr, Ni, Fe_ = 0 μg on Fig. 2 signifies no accumulation. In some patients, the level of measured elements was reduced, which was probably related with individual exposure from other sources. For the majority of patients (41 out of 47 patients for Cr, 34 for Ni, 33 for Fe), an orthodontic appliance was an additional source of exposure to these elements (Fig. 3).

Multiple regression analyses with the use of all statistically significant factors were conducted for Ni and Cr. It was found that, among the selected variables, only sampling as a grouping variable was statistically significant for Cr.

## Discussion

Hair mineral analysis is widely used in the determination of human chronic exposure to various elements, among them toxic metals. Analysis of elements in hair tissue is a non-invasive biomarker of exposure. Sampling and storage of the material is easy and sensitivity of analytical techniques has been recently improved. Although orthodontic treatment is an example of chronic exposure, there is little scientific literature on the subject of hair mineral analysis in the context of metal ions released from orthodontic appliances [14, 18, 19].

The previous results of study by Mikulewicz et al. [14] demonstrated that the content of the following elements was higher in the group of orthodontic patients (*N* = 28) as compared with the control group (*N* = 19): Ni (39 %), Mn (18 %), Fe (4.1 %), and Cr (2.5 %). It was reported that the upper 90th percentile level of the range for Ni was 82.5 % higher for orthodontic patients than for the control patients, suggesting that the orthodontic appliance could be the source of a chronic exposure to nickel. The results were not statistically significant [14].

The reference range as the values between the 10th and 90th percentile were determined for the patients before appliance insertion. These values were used as the normal levels of Cr, Ni, and Fe in hair. The level of these elements in hair of patients during the treatment was referred to the reference values and served as the measure of the extent of exposure. In the present work, the 90th percentile was exceeded in the experimental groups as compared to the values before the treatment (Cr 560–730 %, Ni 16–28 %, Fe 20–87 %). The previous study was carried out on the group of patients vs. a control group consisting of other individuals of the same age. In the present work, the control group individuals were the same persons before the treatment which eliminates the problem of individual differences in hair mineral content or exposure to Ni and Cr from other sources.

In another study, Levrini et al. [18] reported no increase in the content of Ni in hair of orthodontic patients as compared to the control group (0.64 and 0.50 mg/kg in control and experimental group, respectively, *N* = 15). The authors explained that the decrease in the level of Ni in hair of orthodontic patients was caused by significantly higher absorption of nickel from the diet or environmental exposure, as compared with the quantity of nickel ions released from orthodontic appliances.

In the study performed by Abtahi et al. [19], the release of metal ions was investigated in orthodontic patients (24 females) wearing the appliance for 4 months. The control group individuals in this study were not the same patients but their sisters. The content of nickel in hair statistically significantly (*p* = 0.002) increased from 0.382 ± 0.36 mg/kg in the control group to 0.673 ± 0.38 mg/kg in the experimental one. Ni ions were released from the orthodontic appliance and were absorbed, distributed, and eliminated by hair tissue. The concentration of the released nickel ions was higher after appliance placement. The content of nickel in hair of orthodontic patients increased ca. 100 % after 4 months of the treatment. In the present work, after 4 months, the results for Ni were as follows: 0.288 ± 0.334 mg/kg in the control group to 0.366 ± 0.418 mg/kg in the experimental one. The change of the concentration in hair of Ni ions might be the result of different factors: individual differences in absorption and elimination, dietary habits, and environmental exposure. The differences in Ni concentration between II, III, and IV samplings might be associated with the formation of the passivation layer.

Martin-Camean et al. used hair mineral analysis (AAS method) to monitor the release of Cr, Cu, Fe, Mn, and Ni in orthodontic patients (*N* = 70) vs. control (*N* = 56). The content of Cr and Ni remained unchanged. There was no difference between the experimental and the control group: Cr (0.36 ± 0.08 and 0.35 ± 0.26 mg/kg in the control and experimental group, respectively) and Ni (0.47 ± 0.37, 0.46 ± 0.50 mg/kg, respectively). The content of Fe decreased (27.24 ± 17.06 mg/kg, 24.97 ± 15.00 mg/kg) [20]. The control group consisted of different persons, suffering from different environmental and dietary exposure, and having different individual characteristics of hair, which might influence the outcomes. In the present work, the control were the same patients, but before the treatment.

The mean values of Cr, Ni, and Fe in hair of orthodontic patients in our study were compared to the reference ranges found in the literature on the subject and were found to be below the upper limit [21–23]. In the relevant literature, there are no data on the mass, or dose to which orthodontic patients were exposed. In the present work, the sum of the masses of the Cr, Ni, and Fe released to hair was measured within 1 year of therapy. The daily masses were respectively as follows: 0.0245, 0.0203, and 0.778 μg.

The questionnaire survey made it possible to identify confounder factors. The results of the questionnaire survey on the effect of other factors on the level of the investigated elements in hair were analyzed (Table 2). The median values were reported, mean values and SD for the information. It was found that the level of Cr did not depend on sex neither on age. The questionnaire survey covered also other sources of exposure to Cr and Ni (Table 4). This effect was studied on a group before orthodontic treatment. The intake of Coca-Cola increased the level of Cr three times. Drinking tea with lemon caused a higher level of Ni in hair (2.2 times, Table 4). The consumption of yogurt resulted in a 2.4 times higher Ni level (Table 4). All these differences were statistically significant and all these factors were regarded as the second grouping variable. Although there were identified certain dietary factors that caused a higher level of metals in hair samples, this causal does not let itself be analyzed quantitatively.

**Table 4:** The effect of dietary habits on contents of chromium and nickel in hair collected before treatment, mg/kg

		Cr				Ni			
		Mean	SD	Median	*p*	Mean	SD	Median	*p*
Tea with lemon	Yes, *N* = 32	0.0190	0.0423	0.00100	0.882	0.336	0.366	0.239	0.009
	No, *N* = 15	0.0226	0.0460	0.00100		0.150	0.156	0.111	
Coca-Cola	Yes, *N* = 21	0.0321	0.0545	0.00100	*0.095*	0.300	0.410	0.185	0.692
	No, *N* = 26	0.0105	0.0285	0.00100		0.256	0.244	0.221	
Yogurt	Yes, *N* = 38	0.0200	0.0437	0.00100	0.572	0.310	0.353	0.231	*0.068*
	No, *N* = 9	0.0212	0.0426	0.00100		0.131	0.056	0.144	
Allergic to nickel	Yes, *N* = 5	0.00828	0.01627	0.00100	0.641	0.0820	0.0796	0.0832	*0.037*
	No, *N* = 42	0.0216	0.0451	0.00100		0.298	0.336	0.223	

Mann–Whitney *U* test was used. Statistically significant at *p* < 0.1 are presented in italic

Previously, Mikulewicz et al. [14] defined coefficients *α*_hair_ and *β*_hair_ for the evaluation of bioavailability as the quotient (dimensionless value):

$$ {\alpha}_{\mathrm{Hair}}=\frac{C_{\mathrm{With}\ \mathrm{appl}}}{C_{\mathrm{With}\mathrm{out}\ \mathrm{appl}}} $$

and the difference (expressed as mg/kg):

$$ {\beta}_{\mathrm{Hair}}={C}_{\mathrm{With}\ \mathrm{appl}}-{C}_{\mathrm{With}\mathrm{out}\ \mathrm{appl}} $$

where

*C*_*With appl*_: The content of elements (Cr, Fe, Ni) in hair of patients during orthodontic treatment
*C*_*Without appl*_: The content of elements (Cr, Fe, Ni) in hair of patients before the beginning of orthodontic treatment

In the present work, *α* coefficient (dimensionless, showing how many times the content increased vs. control sample) takes the following values after 4, 8, and 12 months of therapy, respectively: for Cr (9.1, 8.9, 7.9), for Ni (1.3, 1.1, 1.5), and for Fe (1.4, 1.3, 1.4). This shows that the content of Cr increased several times while the level of Ni and Fe only to a certain extent. The value of the coefficient was in all cases higher than 1 which signifies that the metal ions released from the appliance were transferred to hair tissue. *β* coefficient (showing the increase in the content as expressed in mg/kg) was as follows: for Cr (0.16, 0.16, 0.14), for Ni (0.078, 0.032, 0.134), and for Fe (4.6, 2.8, 3.9). Since the coefficient *β* reflects the difference in the content of metal ions between the samplings during the treatment and before the treatment, it indicated an increased content of Cr, Ni, and Fe in hair as a result of orthodontic treatment. This difference (expressed in mg/kg) was the highest for Fe because this is the major trace element and is the main component of stainless steel. For all the elements examined, the difference was higher than 0, which shows the transfer of metal ions released from the orthodontic appliance to hair tissue. On the basis of the evaluation of *α* and *β* coefficients, hair mineral analysis seems to reflect the exposure to Cr, Fe, and Ni from orthodontic appliances. This can be observed in Fig. 2, where the baseline reflects *α* = 1 and *β* = 0, which shows no transfer of metals to hair tissue.

## Conclusions

The kinetics of metal ions released from orthodontic appliance and their transfer to hair tissue can be evaluated by a biomarker of chronic exposure, which is hair mineral analysis. The outcomes of the present study revealed that the content of Cr was statistically significantly higher during the treatment than before the beginning of therapy. However, the doses of released metal ions did not pose a toxicological danger.
